# The emerging roles of circular RNAs in ovarian cancer

**DOI:** 10.1186/s12935-020-01367-9

**Published:** 2020-06-23

**Authors:** Xuejing Yang, Jie Mei, Huiyu Wang, Dingyi Gu, Junli Ding, Chaoying Liu

**Affiliations:** Department of Oncology, Wuxi People’s Hospital Affiliated to Nanjing Medical University, No. 299 Qingyang Road, Wuxi, 214023 China

**Keywords:** Circular RNA, Ovarian cancer, Biomarker

## Abstract

Circular RNA (circRNA) is a novel class of regulatory noncoding RNA (ncRNA) molecules with a unique covalently closed loop structure. Next-generation sequencing shows that thousands of circRNAs are widely and stably expressed in multiple eukaryotes. As novel regulatory ncRNAs, circRNAs possess several specific molecular functions, including regulating gene transcription and translation, acting as miRNA sponges, and interacting with functional proteins. Ovarian cancer (OvCa) is one of the most aggressive malignant diseases affecting the lives of thousands of women worldwide, and the majority of OvCa cases are diagnosed at advanced stages. Accumulating evidence has revealed the significant roles of circRNAs in the occurrence and progression of OvCa, indicating the function of circRNAs as promising biomarkers and their therapeutic relevance in this disease. This review aims to summarize the mechanisms by which circRNAs mediate OvCa progression as well as their diagnostic and prognostic values in OvCa.

## Background

Ovarian cancer (OvCa) is one of the most aggressive female malignancies and is estimated to account for approximately 21,750 new cases and 13,940 deaths worldwide in 2020 [1]. OvCa tends to be diagnosed at advanced stages (stage III or IV) with poor prognosis because the ovary is located deep in the abdominal cavity and the early symptoms of OvCa are not obvious [2]. With the development of medical technology, the prognosis of OvCa has significantly improved, but the 5-year overall survival (OS) rate remains low, between 35 and 40% [3]. Hence, discovery of the molecular biological mechanism of OvCa tumorigenesis and development has captured extensive attention from researchers, and novel OvCa-associated biomarkers may be essential for enhancing the diagnosis and prognosis of these patients.

Noncoding RNAs (ncRNAs), which constitute more than 98% of the human genome, play essential roles in gene regulation and translation. Importantly, a growing number of studies have proven the role of ncRNAs in tumorigenesis, including cell proliferation, apoptosis, migration, and invasion [4, 5]. CircRNA is a class of recently re-recognized ncRNA with a distinct structure, which has not been well studied and was originally regarded as the result of mis-splicing or as a byproduct of pre-mRNA processing with low abundance over the past 30 years [6]. Currently, the rapid expansion of next-generation sequencing, extranuclear enrichment tools, and bioinformatics analyses have confirmed the critical roles of circRNAs in the pathogenesis of a large number of human diseases, including heart diseases [7], diabetes [8], nervous system diseases [9], immune system diseases [10] and cancers [11]. In addition, increasing numbers of studies have shown that circRNAs may be involved in cancer proliferation, invasion, metastasis, and apoptosis, indicating the use of circRNAs as promising biomarkers and their therapeutic relevance [12–14]. In particular, circRNAs may serve as essential regulatory factors in OvCa occurrence and progression according to recent publications [11, 15, 16]. Thus, the current review discusses the dysregulated expression of circRNAs and circRNA-mediated OvCa progression as well as the diagnostic and prognostic values of circRNAs in OvCa.

## Characteristics of circRNAs

CircRNAs, differing from linear RNAs, are a class of re-recognized ncRNAs with covalently closed structures that were first reported in the viroid genome by Sanger et al. in 1976 [17]. In recent years, scholars have found that circRNAs are present in a stable structure in various types of eukaryotic cells, including in protists, fungi, plants, fish, worms, insects, and mammals [18]. CircRNAs often share the following characteristics: (1) circRNAs are characterized by their covalently closed loop in which the 5′ and 3′ ends are connected to each other; (2) circRNAs are prone to be highly resistant to endonuclease cleavage in that they have no exposed 5′ or 3′ end, and circRNAs are more stable due to their closed structure in comparison with their linear counterparts [19]; (3) circRNAs are generally expressed in eukaryotes, and their expression levels vary extremely in a tissue-specific and developmental-stage-specific manner. For example, Ahmed et al. [20] examined the differential expression of circRNAs in primary and metastatic OvCa tissues; (4) circRNAs are mainly derived from protein-coding genes, and one gene may generate various circRNAs through selective circularization [21]; (5) most circRNAs are evolutionarily and sequentially conserved in various species [22]; and (6) circRNAs could be divided into three subtypes, namely, exonic circRNAs, composed of only exons, are primarily found in the cytoplasm; intronic circRNAs, originating from introns, are principally present in the nucleus; and exon–intron circRNAs, which contain both introns and exons and are mainly present in the nucleus [23]. Overall, conservation, specificity, and stability are the three major characteristics of circRNAs. These features indicate that circRNAs potentially play functional roles in gene expression at the transcriptional and posttranscriptional levels and may therefore influence diagnoses, treatment, and prognoses of numerous diseases.

## Biogenesis of circRNAs

In eukaryotic genes, pre-mRNA undergoes alternative splicing, thus removing the noncoding introns and reattaching the exons [24]. After that, the noncoding introns that are removed by the splicing process can be shaped into linear or lariat molecules. Accumulating evidence has demonstrated that circRNAs are generated with the aid of RNA polymerase II (Pol II) in the splicing process [23]. On the basis of RNA composition and biogenesis mechanisms, circRNAs can be grouped into three subclasses: exonic circRNAs (ecircRNAs), circular intronic RNAs (ciRNAs), and exon–intron circRNAs (EIciRNAs) [23]. EcircRNAs, which contain only exons, are generated in a process called back-splicing circularization or exon skipping event [25]. Back-splicing circularization, also called circle splicing or head-to-tail splicing, is a process where the downstream 5′ end of an exon binds to the upstream 3′ end of another exon, giving rise to a circular RNA. A single exon may also generate a circular RNA; in this case, the 3′ end of one exon is linked to the 5′ end of the same exon [18]. Exon skipping is the other process of generating ecircRNAs, where pre-mRNAs first undergo exon skipping and then form an exon-containing lariat with the aid of looped introns during internal splicing [26].

**Fig. 1:**
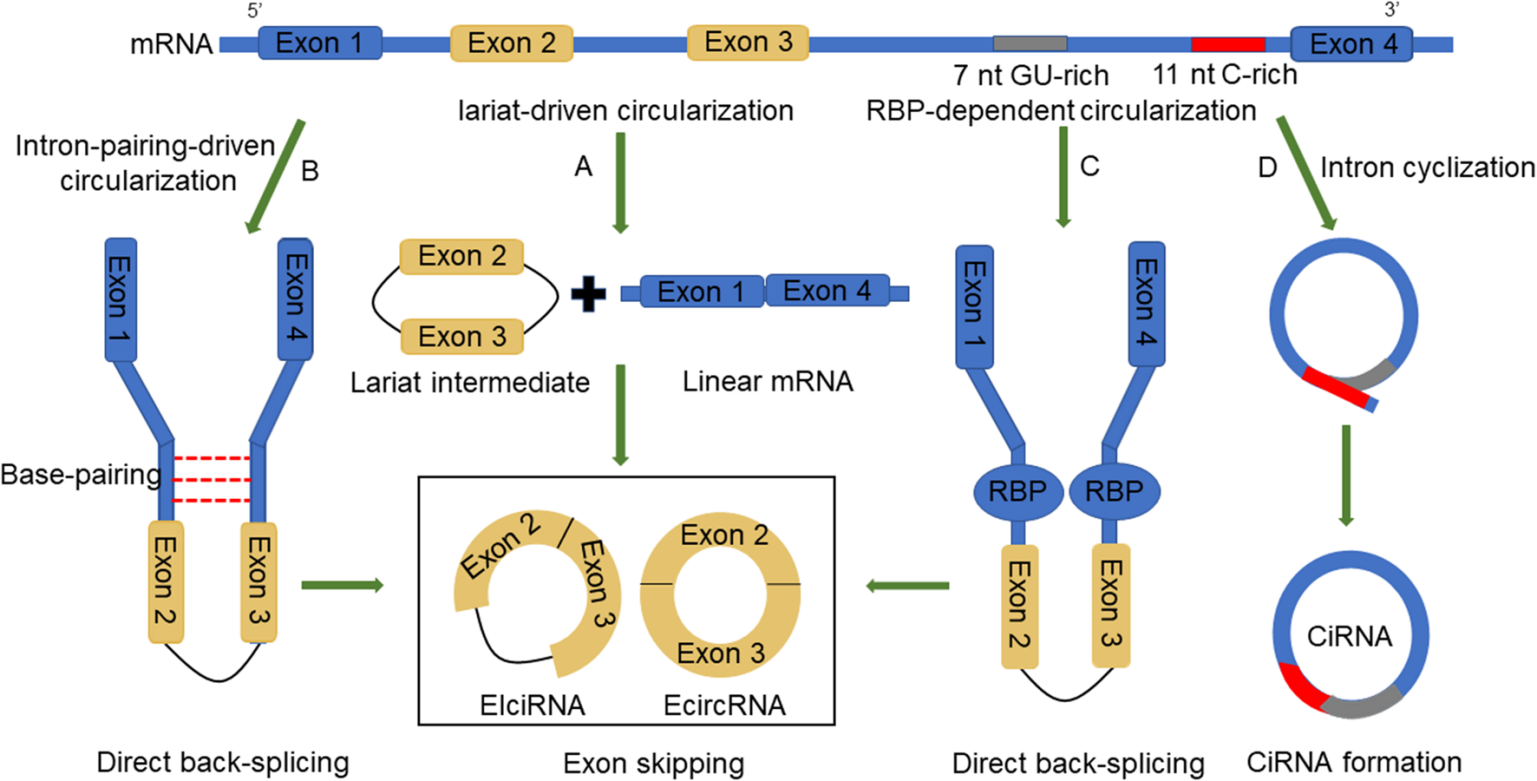
Biogenesis of circRNAs: four models explain circRNA formation. **a** Lariat-driven circularization first generates a lariat intermediate in which the 5′ end donor of an exon binds to the 3′ end acceptor of another exon and subsequently forms an ecircRNA by removing the introns between the exons. **b** Intron pairing-driven circularization begins with cyclization with the aid of reverse complementary sequences and thus generates an EIciRNA or ecircRNA via intron removal. **c** RBP-driven circularization starts with back-splicing, and then the flanking introns bind to each other closely with the aid of RBPs, resulting in the formation of a circRNA. **d** CiRNA formation is dependent on the conserved sequence near both sides of the spliceosome. The conserved sequence assists the intronic lariat to evade degradation by debranching enzymes

Lariat-driven circularization, intron pairing-driven circularization, and RNA binding protein (RBP)-dependent circularization are the major models to explain the formation of ecircRNA or EIciRNA. Lariat-driven circularization, an exon skipping process, first generates a lariat intermediate in which the 5ʹ end donor of an exon binds to the 3ʹ end acceptor of another exon and subsequently forms an ecircRNA by removing the introns between the exons [22] (Fig. 1a). Intron pairing-driven circularization, a direct back-splicing mechanism, is a process that induces cyclization with the aid of reverse complementary sequences and thus generated an EIciRNA or ecircRNA via intron removal [21] (Fig. 1b). RBP-driven circularization, the other model of the back-splicing mechanism, starts with back-splicing, and then the flanking introns bind to each other closely with the aid of RBPs, resulting in the formation of a circRNA [27] (Fig. 1c). However, unlike ecircRNA, ciRNA formation is dependent on a conserved sequence near both sides of the spliceosome [28]. When a 3′ splice acceptor binds to a 5′ splice donor, thus generating a lariat intron, the conserved sequence composed of a 7 nt GU-rich element close to the 5′ splice site and an 11 nt C-rich element near the branchpoint site helps the intronic lariat evade degradation by debranching enzymes [29] (Fig. 1d).

In conclusion, the biogenesis of circRNAs has not been completely elucidated, and the mechanism described above represents only a limited point of view. In future years, by the use of advanced computing tools, it is likely that the mechanism of circRNA biogenesis will be further better understood.

## Functions of circRNAs

### CircRNAs act as ceRNAs or miRNA sponges

**Fig. 2:**
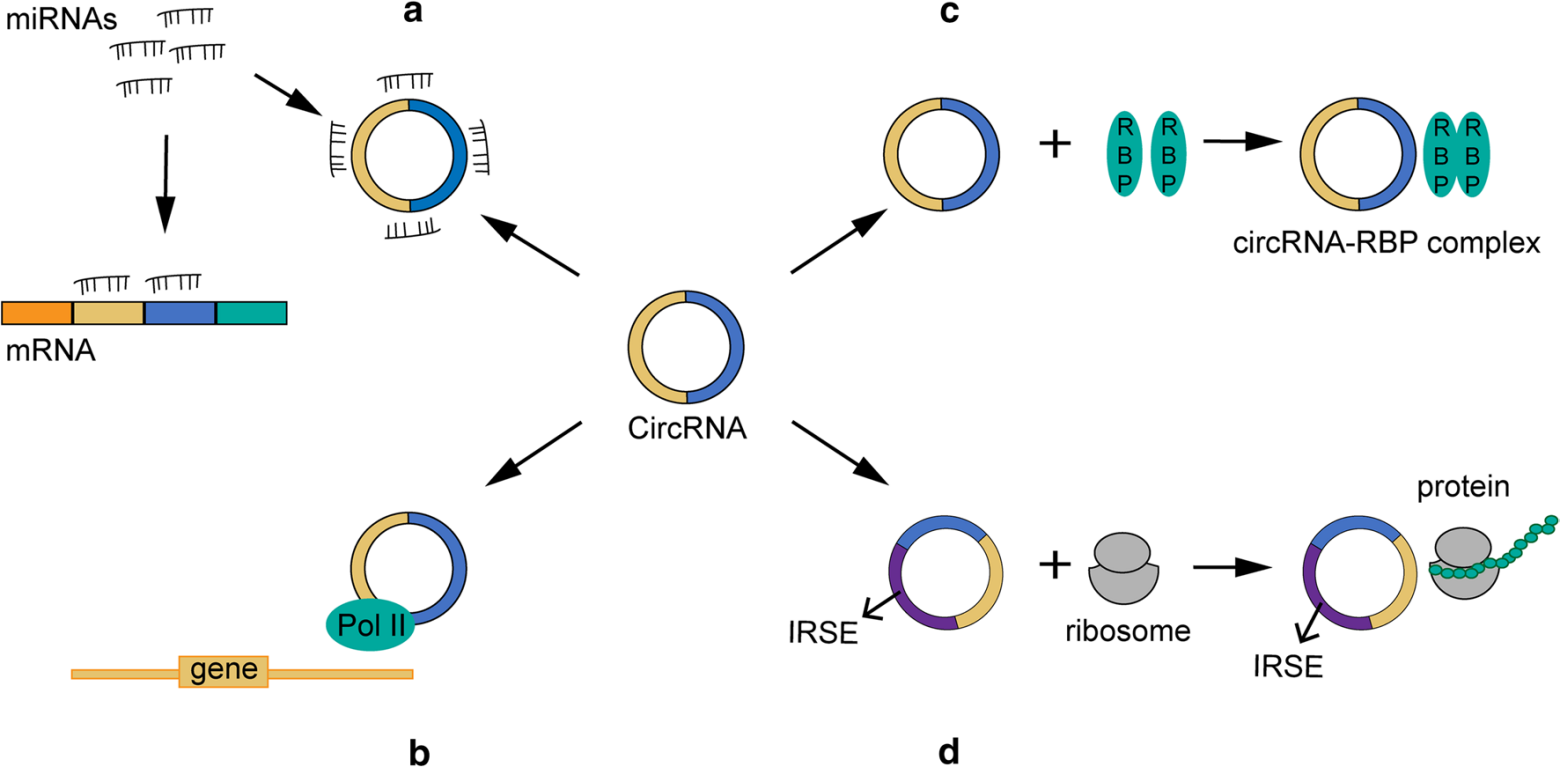
Functions of circRNAs. **a** miRNA sponges; **b** Regulation of gene transcription; **c** Interaction with functional proteins; **d** Translation into peptides and/or proteins

Multiple studies have uncovered the function of circRNAs as competing endogenous RNAs (ceRNAs) or miRNA sponges, whereby the indirectly regulate gene expression by competing for miRNA binding (Fig. 2a). CeRNAs are transcripts that possess miRNA response elements (MREs) and include long noncoding RNAs (lncRNAs), mRNAs, pseudogenic RNAs, and circRNAs, among which circRNAs have the highest binding capacity to miRNAs. It is no exaggeration to say that circRNA has become a novel research hotspot in the ceRNA field due to its unique characteristics and has attracted tremendous attention from scholars [30, 31]. The ceRNA hypothesis indicates that circRNA shares a similar seed sequence with the target mRNA, thus sponging miRNAs that regulate the function and expression levels of the target gene [32]. EcircRNAs, which contain MREs with a stable circular structure, are reported to act as functional ceRNAs that possess the capability to bind to miRNAs and prevent their binding to target mRNAs [32]. It has been reported that several types of malignant diseases are associated with an aberrant circRNA–miRNA–mRNA axis. For instance, CiRS-7, which possesses 73 miR-7 binding sites, can sponge miR-7, thus inhibiting the biological function of miR-7 target genes. It has been confirmed that CiRS-7 mediates apoptosis by sponging miR-7 to regulate the PTEN/PI3K/AKT pathway in gastric cancer [33]. In addition, circ-ITCH is reported to increase the cancer-suppressing ability of its parental gene by sponging miR-7 and miR-214 and consequently attenuating lung cancer cell proliferation [34]. Hsa_circ_0012673 promotes cell proliferation in lung cancer tissues and cells through regulation of LIM domain kinase 1 (LIMK1) levels by sponging miR-320a [15]. In conclusion, circRNAs may commonly exert their regulatory functions in cancers by acting as miRNA sponges. However, to date, scholars have characterized only a small fraction of these circRNAs as well as their sponge functions, and extensive research is needed to explore the rest of the circRNA family.

### CircRNAs regulate gene transcription

In addition to acting as sponges for miRNAs, a portion of circRNAs can *cis*- or *trans*-regulate gene transcription, thus impacting gene expression [35] (Fig. 2b). CiRNAs, primarily located in the nucleus, are reported to modulate the transcription and expression of their parental genes by binding to RNA Pol II [28]. Typical examples are ci-ankrd52 and ci-sirt7. It has been confirmed that both circRNAs can positively regulate the transcription of their parental genes by interacting with Pol II [28]. Additionally, EIciRNAs are also thought to regulate gene expression. For instance, Li et al. found that both EIciRNAs, circ-EIF3J, and circ-PAIP2, can increase the expression of their parental genes in HEK293 and HeLa cells via their involvement in the interaction between U1 small nuclear ribonucleic proteins (snRNPs) and RNA Pol II in the promoter region of the host gene [36]. In summary, circRNAs can function as regulator factors to impact gene expression by binding with the RNA pol II complex and transcription-related proteins.

### CircRNAs interact with functional proteins

Although most studies have focused on circRNA sponge activity, several studies have demonstrated that circRNAs harbor numerous protein-binding sites for RBPs and exert biological functions by binding to RBPs (Fig. 2c). For example, CircFoxo3, which is mainly expressed in the cytoplasm, was proven to interact with the anti-senescence proteins ID1 and E2F1 and the anti-stress proteins FAK and HIF1a, arresting them in the cytoplasm and preventing them from moving to the nucleus, thus inhibiting their antisenescence and anti-stress functions [37]. In addition, circFoxo3 was reported to bind to the cell cycle proteins cyclin-dependent kinase 2 (CDK2) and cyclin-dependent kinase inhibitor 1 (p21), giving rise to a circ-Foxo3-p21-CDK2 ternary complex, thus arresting the cell cycle and blocking the transition from G1 to S phase [38]. Yang et al. [39] detected a circRNA, derived from angiomotin-like 1, which was colocalized with an oncogenic protein called c-myc in the nucleus, thus preventing the degradation of this protein and promoting the tumorigenesis of breast cancer. Overall, circRNAs could exert biological functions by interacting with cancer-associated proteins, suggesting their potential as novel promising targets in cancer therapy.

### CircRNAs can be translated into proteins

CircRNAs are regarded as a type of distinct noncoding RNAs that cannot be translated into peptides due to their closed loop structure and lack of polyadenylated tail and internal ribosome entry site (IRES). In contrast, the majority of circRNAs are derived from protein-coding genes and contain complete exons, indicating the potential of circRNAs to be translated into polypeptides (Fig. 2d). To date, a portion of circRNAs has been proven to share this function. For example, Chen et al. [40] found that after inserting an IRES into a synthetic circRNA, circRNAs were able to bind to the eukaryotic ribosome and initiate the translation process both in vitro and in vivo. Additionally, it has been confirmed that viral circRNAs can be translated into proteins in eukaryotes. A typical example is circRNAs in hepatitis D virus (HDV), which possess an IRES and can be translated into the hepatitis D virus antigen (HDAg) after infection of human cells [41]. CircZNF609 is another circRNA that is proven to translate protein, providing a novel example of protein-coding circRNA in eukaryotic cells [42]. This circRNA possesses an open reading frame (ORF) with a start codon and a stop codon on both sides and can generate a protein due to the detected structure [42]. Besides, inserting an artificial circRNA into the green fluorescent protein (GFP) ORF could result in the production of extremely long protein chains in *Escherichia coli* [43]. Additionally, studies by Pamudurti et al. discovered that circMbl3 could be translated into polypeptides in fly heads [44].

As mounting evidence has determined the function of circRNAs as protein-coding RNAs, an increasing number of studies are focused on the specific mechanism of circRNA being translated into peptides. For example, Naoko et al. indicated that circRNAs possessing an infinite ORF can produce long protein chains via a rolling cycle amplification mechanism [45, 46]. In addition, studies of Yang et al. demonstrated a common mechanism of circRNAs being translated in a m6A-driven manner. N6-methyladenosine (m6A), one of the most abundant modifications in ncRNAs, was proved to facilitate translation initiation of circRNAs via acting as IRESs and researchers investigated that such a single modification is sufficient to initiate translation [47].

## CircRNAs in OvCa

Currently, circRNAs have gradually become a novel research hotspot among ncRNAs and have subsequently captured the attention of the cancer research field. Numerous studies have demonstrated that circRNAs are differentially expressed in a variety of cancerous tissues. Moreover, an increasing number of studies have proven the presence of aberrantly expressed circRNAs in OvCa tissues, suggesting the relevance of circRNAs in the tumorigenesis and progression of OvCa. We discuss the potential diagnostic and prognostic values of circRNAs in OvCa and summarize the biological and cellular functions of several representative circRNAs in OvCa (Table 1).

**Table 1:** Summarization of the cellular functions of circRNAs in tumorigenesis of OvCa

CircRNAs	Circbase ID	Role in OvCa	Cancer phenotypes	Sponge miRNAs	Target genes	References
circMUC16	hsa_circ_0049116	Oncogene	Promoted proliferation and invasion	miR-199a-5p	Beclin1/RUNX1	[55]
circCSPP1	hsa_circ_0001806	Oncogene	Promoted proliferation, migration, invasion and EMT	miR-1236-3p	ZEB1	[58]
circEPSTI1	hsa_circ_0000479	Oncogene	Promoted proliferation, invasion and induced apoptosis	miR-942	EPSTI1	[61]
/	hsa_circ_0051240	Oncogene	Promoted proliferation, migration, and invasion	miR-637	KLK4	[62]
circWHSC1	hsa_circ_0001387	Oncogene	Promoted proliferation, migration, invasion, EMT and induced apoptosis	miR-145/miR-1182	MUC1/hTERT	[64]
/	hsa_circ_0061140	Oncogene	Promoted proliferation, invasion and EMT	miR-370	FOXM1	[16]
circHIPK3	hsa_circ_0000284	Tumor suppressor	Inhibited proliferation, migration and invasion and promoted apoptosis	miR-10a-5p/miR-148b	/	[77]
circLARP4	has_circ_101057	Tumor suppressor	Inhibited proliferation and migration	miR-513b-5p	LARP4	[81]
circPLEKHM3	hsa_circ_0001095	Tumor suppressor	Inhibited proliferation and migration	miR-9	BRCA1/NAJB6/KLF4	[82]
CircITCH	/	Tumor suppressor	Inhibited proliferation, migration, invasion and promoted apoptosis	miR-10a-α/miR-145	RASA1	[83, 84]

### CircRNAs function as diagnostic and prognostic biomarkers

Most functional circRNAs tend to exhibit differential expression patterns between OvCa patients and healthy individuals. Furthermore, circRNAs can reflect cancer progression and can be used to monitor the prognosis of OvCa patients, and thus have the potential to act as biomarkers in clinical practice.

Circulating cell-free circRNAs extracted from blood is a hotspot in the field of diagnostic biomarkers. According to published literature, circBNC2 could be an OvCa-specific diagnostic biomarker distinguishing early-stage OvCa patients from benign and healthy cohorts [48]. Circulating circABCB10 and circMAN1A2 also acted as diagnostic biomarkers for OvCa [49, 50]. Furthermore, Sheng et al. identified the circRNA profiles in OvCa specimens and demonstrated that circBNC2, circEXOC6B, circFAM13B, circN4BP2L2, circRHOBTB3, and circCELSR1 were notably dysregulated in OvCa tissues and may be promising diagnostic biomarkers for OvCa [51].

Since the prognosis of OvCa patients is quite poor, prognostic evaluation is essential for both clinicians and patients. Positive circABCB10 expression was significantly associated with poor OS in OvCa patients [50]. Low expression of circEXOC6B and circN4BP2L2 exhibited a tight association with OS and PFS in OvCa patients [51]. In addition, circRNA1656 was reported to be downregulated in both high-grade ovarian serous carcinoma (HGSOC) tissue and OvCa cell lines and correlated with the International Federation of Gynecology and Obstetrics (FIGO) stage of HGSOC, suggesting its potential to serve as a novel tumor marker for HGSOC [52].

On the other hand, several circRNAs have been shown to function as tumor suppressors in OvCa and serve as prognostic indicators. CircHIPK3 expression was higher in OvCa tissues than in matched adjacent noncancerous tissues, and higher circHIPK3 expression was positively associated with lymph node invasion, advanced FIGO stage, and worse DFS and OS in OvCa patients [53]. In addition, circLARP4 was proposed to decrease in OvCa tissues compared to adjacent normal tissues, and its lower expression was positively associated with advanced tumor FIGO stage, lymph node metastases, and worse disease-free survival (DFS) and OS [54].

### CircRNAs act as tumor promoters

#### Hsa_circ_0049116

Hsa_circ_0049116, also named circMUC16, is spliced from the MUC16 gene. The expression of this circRNA was significantly elevated in OvCa tissues and serum of patients with OvCa. Moreover, highly expressed circMUC16 was reported to be positively associated with tumor stage and tumor grade. Gan et al. revealed that circMUC16 mediated autophagic flux in OvCa by regulating the circMUC16-miR-199a-5p-Beclin1/RUNX1 axis, thereby accelerating the proliferation and invasion of OvCa cells [55]. In addition, several studies have revealed that transcription factors can modulate the expression of circRNAs, and circMUC16 is a typical example. The expression of this circRNA was reported to be impacted by the downstream target runt-related transcription factor 1 (RUNX1) via transcriptional promotion. Gan et al. discovered that overexpression of RUNX1 facilitated MUC16 promoter activity and increased the expression of circMUC16, while silencing RUNX1 inhibited MUC16 promoter activity and decreased circMUC16 expression [55].

#### Hsa_circ_0001806

Hsa_circ_0001806, also called circCSPP1, is generated from the centrosome/spindle pole-associated protein 1 (CSPP1) gene. CSPP1, an oncogene located in the 8q13.2 region of the human genome, has been thoroughly investigated in luminal breast cancer and B-cell lymphoma [56, 57]. Additionally, circCSPP1 was found to be preferentially upregulated in borderline and tumor compared to benign and cancer specimens, and the expression of this circRNA was positively correlated with stage II–V [58]. Subsequently, Li et al. demonstrated that knockdown of circCSPP1 suppressed cell proliferation, migration, and invasion, while circCSPP1 overexpression promoted cancer behavior. In addition, miR-1236-3p, a tumor suppressor in OvCa, was negatively correlated with circCSPP1 in OvCa cell lines [58]. MiR-1236-3p was able to suppress cancer properties by targeting zinc finger E-box binding homeobox1 (ZEB1) in OvCa, while circCSPP1 was reported to sponge miR-1236-3p and suppress the silencing effect on ZEB1, thereby stimulating epithelial-to-mesenchymal transition (EMT) and cancer progression in OvCa [58, 59]. Moreover, Li et al. revealed that circCSPP1 exerted a positive impact on the expression of oncogenic proteins in OvCa, including vascular endothelial growth factor A (VEGFA) and matrix metalloproteinase-2 (MMP2) [58].

#### Hsa_circ_0000479

Hsa_circ_0000479, also named circEPSTI1, was reported to be upregulated in triple-negative breast cancer (TNBC) specimens, and the high expression of circEPSTI1 was related to decreased survival in these cases. It was demonstrated that circEPSTI1 was able to facilitate cell growth and suppress apoptosis by binding to miR-4753 and miR-6809 to upregulate BCLI1A expression in TNBC [60]. Recently, an increasing number of studies have confirmed the critical role of circEPSTI1 in OvCa development. CircEPSTI1 was remarkably upregulated in OvCa specimens compared to adjacent nontumor counterparts. In addition, circEPSTI1 was correlated with tumor size and lung metastasis in mouse xenografts, and this circRNA may potentially enhance OvCa cell proliferation and invasion and inhibit cell apoptosis in vivo [61]. Moreover, miR-942, a tumor suppressor in OvCa, was found to directly target the linear form of circEPSTI1, while circEPSTI1 was proven to sponge miR-942, suggesting a potential effect of circEPSTI1 on EPSTI1, which was downregulated in OvCa tissues [61].

#### Hsa_circ_0051240

Hsa_circ_0051240, derived from the gene CEACAM5, is another circRNA that is upregulated in OvCa. This highly expressed circRNA was reported to enhance cell proliferation, invasion and migration by targeting miR-637 to regulate the expression of Kallikrein-related peptidases 4 (KLK4), and KLK4 was reported to be involved in many steps of OvCa tumorigenesis and progression and to exert modulatory effects on a subset of cancer-related genes and proteins in several cancers [62, 63].

#### Hsa_circ_0001387

Hsa_circ_0001387, also called circWHSC1, is derived from the WHSC1 gene through the exonic back-splicing process. This circRNA was confirmed to function as an oncogenic circRNA and may represent a novel promising biomarker and therapeutic strategy for OvCa. Zong et al. found that circWHSC1 was highly expressed in OvCa and was able to adsorb miR-145 and miR-1182 to upregulate the expression of downstream targets Mucin 1 (MUC1) and human telomerase reverse transcriptase (hTERT) [64]. MUC1 is a highly expressed gene in a variety of cancers, including OvCa, and is known to participate in tumorigenicity, initiate the EMT process, and coordinate cancer metastasis [65–67]. HTERT was found to be essential in cancer cell behavior, and ectopic expression of this oncogene could induce EMT by upregulating Slug [68, 69]. Importantly, circWHSC1 can be secreted into exosomes. Zong et al. claimed that this circRNA can be absorbed by peritoneal mesothelial cells in the form of exosomes and facilitates EMT changes, thus triggering tumor distribution in the peritoneal cavity and promoting the development of OvCa [64].

#### Hsa_circ_0061140

Chen et al. demonstrated that hsa_circ_0061140 was significantly upregulated in OvCa cell lines using real-time qPCR. Subsequent experimental data suggested that hsa_circ_0061140 silencing caused an arrest in cell proliferation and invasion as well as EMT in SKOV3 and A2780 cells by sponging miR-370 to inhibit the expression of forkhead box protein M1 (FOXM1) [16]. MiR-370 is known to be decreased in OvCa and acts as an anticancer gene. Overexpression of miR-370 was reported to inhibit cell growth and invasion in a variety of cancers, including breast cancer [70], bladder cancer [71], and hepatocellular carcinoma [72], while silencing of hsa_circ_0061140 was reported to increase miR-370 expression. Bioinformatics analyses revealed that FOXM1, a transcription factor that is involved in the cell cycle and cell proliferation, may potentially be the downstream target of miR-370 [16]. Therefore, overexpression of FOXM1 was detected to rescue miR-370-mediated inhibition of migration by wound healing and transwell analyses and rescue miR-370-induced EMT inhibition by western blot [16]. Overall, hsa_circ_0061140 upregulated FOXM1 expression by binding to miR-370, providing a promising therapeutic target for OvCa.

### CircRNAs act as tumor suppressors

#### Hsa_circ_0000284

Hsa_circ_0000284, also called circHIPK3, is localized primarily in the cytoplasm of OvCa cells and was found to exhibit different functions in different cells or tissues. Zheng et al. and Shan et al. discovered that knockdown of circHIPK3 could inhibit the proliferation of retinal endothelial cells and HEK-293 T cells [73, 74]. Zeng et al. claimed that silencing circHIPK3 inhibited cell invasion and migration and promoted apoptosis in colorectal cancer [75]. In contrast, Li et al. found that overexpression of this circRNA inhibited cell migration and invasion in bladder cancer [76].

Interestingly, the concrete function of circHIPK3 in OvCa varies extremely. In the study by Teng et al., qRT-PCR and the expression profile of circHIPK3 both confirmed that this circRNA was decreased in epithelial OvCa cell lines compared with normal ovarian tissue cells, while Liu et al. discovered that the expression of this circRNA was significantly increased in OvCa tissues compared to their matched adjacent noncancerous counterparts [53]. The same study by Teng et al. revealed that in both normal ovarian epithelial cells (IOSE80) and OvCa cells (A2780 and SKOV3) silencing of circHIPK3 could facilitate OvCa processes, including cell proliferation, migration, and invasion, and inhibited cell apoptosis, providing a novel example of cell-type-specific expression and function of circRNA [77].

Additionally, a growing number of studies have concentrated on the function of circHIPK3 as a miRNA sponge. For instance, circHIPK3 has been shown to sponge miR-30a-3p in diabetes mellitus, miR-558 in bladder cancer cells, miR-7 in colorectal cancer, and miR-124 in HeLa cells [73–76]. It was reported that this circRNA was capable of sponging miR-193-3p to restore MCL1 expression, which was remarkably expressed in numerous cancers [78]. In the study by Teng et al., circHIPK3 was found to possess 1–3 binding sites each of 12 miRNAs in OvCa, namely, miR-224-3p, miR-579-3p, miR-10a-5p, miR-148b-3p, miR-876-5p, miR-193a-3p, miR-30d-3p, miR-499a-5p, miR-522-3p, miR-1278, miR-106a-3p and miR-30b-3p [77]. Moreover, a miR-106a inhibitor was demonstrated to restore OvCa cell proliferation in xenograft mice [79]. MiR-148b was overexpressed in 92.21% of OvCa tissues and might exert a significant function in the tumorigenesis and progression of OvCa [80]. Therefore, circHIPK3 might exhibit its biological function in the tumorigenesis of OvCa by sponging one or several of the 12 miRNAs.

#### Hsa_circ_101057

Hsa_circ_101057, also termed circLARP4, is derived from exons 9 and 10 within the La ribonucleoprotein domain family member 4 (LARP4) locus, which is located on chromosome 12q13.12. This circRNA may serve as a tumor suppressor in OvCa. Additionally, Lin et al. discovered that circRNA expression was significantly decreased in OvCa cell lines compared with the normal group. Additionally, circLARP4 could upregulate its host gene LARP4 posttranscriptionally by sponging miR-513b-5p, thereby inhibiting cell proliferation and migration [81]. Overall, circLARP4 suppressed the development of OvCa via the miR-513b-5p/LARP4 axis, providing a promising therapeutic target for OvCa treatment. However, further clinical investigations with larger samples are needed to confirm the suppressive function of this circRNA.

#### Hsa_circ_0001095

Hsa_circ_0001095, also called circPLEKHM3, is spliced from exons of PLEKHM3 on the reverse strand of chromosome 2. CircPLEKHM3 was remarkably downregulated in OvCa tissues and predicted poor prognosis. Mechanistically, circPLEKHM3 inactivated the AKT1 and canonical Wnt/β-catenin signaling pathways by sponging miR-9 to modulate the expression of KLF4, DNAJB6, and BRCA1, thus suppressing OvCa cell proliferation and migration. Interestingly, Zhang et al. suggested that the combination of MK-2206 and Taxol exhibits a synergetic therapeutic effect on OvCa patients, and this synergetic treatment was elevated in OvCa cells with a loss of circPLEKHM3 expression, providing a novel example of circRNA involved in drug treatment cases [82].

#### CircITCH

Several studies have shown that miR-10a, a noncoding miRNA located on chromosome 17, is capable of promoting the progression of several cancers, and emerging studies have focused on the function of this miRNA in OvCa. Luo et al. detected a decrease in circITCH expression in human OvCa cell lines compared to normal human ovarian epithelial cell lines and determined the effect of circITCH on miR-10a levels. Overexpression of circITCH significantly suppressed cell growth and promoted cell apoptosis via direct interaction with miR-10a [83]. Additionally, Hu and colleagues claimed that circITCH acted as a ceRNA to interact with miR-145, upregulated the level of RASA1, and inhibited the cancerous properties of OvCa cells by regulating the circITCH-miR-145-RASA1 axis in vitro and in vivo [84]. Hence, circITCH may inhibit the malignant progression of OvCa by targeting different miRNAs. However, further investigations are needed to identify the specific downstream gene affected by circITCH acting as a miRNA sponge.

## Future perspective

Accumulating studies have confirmed the critical function of circRNAs in the pathogenesis of numerous diseases. More recently, functional peptides produced by circRNAs have emerged into scientific attention. For example, SHPRH-146aa, encoded by circ-SHPRH, inhibits progression of central nervous system cancer via regulating protein ubiquitination pathways to protect the quality of SNF2 histone linker PHD RING helicase (SHPRH) gene [85, 86]. AKT3-174aa, another tumor-related functional peptide encoded by circ-AKT3, was less expressed in glioblastoma in comparison with normal brain tissues and was proved to suppress cancer properties through negatively regulating the PI3K/Akt signaling pathway in glioblastoma [87]. Evidence have certificated the existence and function of circRNA encoded peptides, however, the systematic detection of such peptides remains difficult due to the technical challenges as well as the similarity between circRNA-encoded peptides and the counterpart of mRNA. Therefore, it is essential to conduct in-depth researches on circRNA-encoded peptides including the specific mechanism of such peptides involving in the pathogenesis of diseases, whether such peptides undergo modification post-translationally as well as the associated condition and factors regulating the translation of circRNAs.

Also, the function of circRNAs in the tumor microenvironment (TME) have been studied, including regulating tumor immune surveillance via serving as immune system antigens [88], mediating immune escape via circRNA-miRNA-PD-1/PD-L1 axis [89], regulating the cytotoxicity of natural killer cells by acting as miRNA sponges and modulating associated proteins [90], promoting or inhibiting angiogenesis through multiple mechanisms [91] and inhibiting the permeability of endothelial cell [92]. Initial results have demonstrated the impact of circRNAs on TME, however, researches in correlation with this topic are in its infancy and further researches need to identify the clinical relevance on this topic.

As well known, hypoxia plays an essential role in cancer aggressiveness and recent researches have focused on the correlation between hypoxia and circRNAs [93]. Several circRNAs have been reported to be impacted by hypoxia including circDENND4C [94], circDENND2A [95], circ-0000977 [96], and circ-0010729 [97]. Detecting the mechanism of circRNAs in hypoxia conditions may provide a new insight into the potential utilities of circRNAs.

## Conclusion

It is no exaggeration to say that circRNAs have become a novel frontier in cancer research and have captured extensive attention from researchers. OvCa-associated circRNAs exert their biological functions in OvCa principally by regulating cell proliferation, invasion, migration, apoptosis, EMT, and cell cycle. Currently, an increasing number of circRNA transcriptomes in OvCa have been revealed, and a few circRNAs have been identified as functional ncRNAs associated with clinical performance. Notably, the characterized cancer-related circRNAs have been shown to play significant roles in the tumorigenesis and progression of OvCa and display superiority in clinical application due to their advantages in diagnosis, prognosis, and therapy. However, the number of experimental samples used to investigate the expression profile and specific function of circRNAs has been relatively small. Therefore, it is urgent to conduct comprehensive studies in a larger number of specimens to confirm the transcriptome sequencing data and functions of circRNAs in OvCa. In particular, the current research mainly revealed the function of circRNAs as miRNA sponges, suggesting that further investigations are needed to explore other potential mechanisms of circRNAs in the pathogenesis of OvCa.

## Data Availability

All data are included in the article.

## Abbreviations

circRNA: Circular RNA
ncRNAs: Noncoding RNAs
OvCa: Ovarian cancer
OS: Overall survival
Pol II: Polymerase II
ecircRNA: Exonic circRNA
ciRNA: Circular intronic RNA
EIciRNAs: Exon–intron circRNA
RBP: RNA binding protein
ceRNA: Competing endogenous RNA
MREs: miRNA response elements
lncRNA: Long noncoding RNA
LIMK1: LIM domain kinase 1
snRNPs: Small nuclear ribonucleic proteins
CDK2: Cyclin-dependent kinase 2
p21: Cyclin-dependent kinase inhibitor 1
IRES: Internal ribosome entry site
HDV: Hepatitis D virus
HDAg: Hepatitis D virus antigen
ORF: Open reading frame
GFP: Green fluorescent protein
m6A: N6-methyladenosine
HGSOC: High-grade ovarian serous carcinoma
FIGO: International Federation of Gynecology and Obstetrics
DFS: Disease-free survival
RUNX1: Runt-related transcription factor 1
EMT: Epithelial-to-mesenchymal transition
VEGFA: Vascular endothelial growth factor A
MMP2: Matrix metalloproteinase-2
TNBC: Triple-negative breast cancer
KLK4: Kallikrein-related peptidase 4
MUC1: Mucin 1
hTERT: Human telomerase reverse transcriptase
FOXM1: Forkhead box protein M1
LARP4: La ribonucleoprotein domain family member 4
SHPRH: SNF2 histone linker PHD RING helicase
TME: Tumor microenvironment
